# Incidence of seropositive rheumatoid arthritis in population-based studies in Northern Savo, Finland, during 1980–2020

**DOI:** 10.1007/s00296-022-05268-0

**Published:** 2023-01-11

**Authors:** Pia Elfving, Aulikki Kononoff, Johanna Huhtakangas, Hannu Kautiainen, Elina Savolainen, Leena Arstila, Julia Barantseva, Tuomas Rannio, Helena Niinisalo, Antti Puolitaival, Kati Soininen, Simo Kariniemi, Minni Oksaranta, Oili Kaipiainen-Seppänen

**Affiliations:** 1 Department of Medicine, Kuopio University Hospital, PO. Box 100, 70029 KYS Kuopio, Finland; 2 Institute of Clinical Medicine, University of Eastern Finland, Kuopio, Finland; 3 Iisalmi Hospital, Iisalmi, Finland; 4 Pihlajalinna, Kuopio, Finland; 5 Mehiläinen, Kuopio, Finland; 6 Department of Public Health, Kuopio University Hospital, Kuopio, Finland; 7 Folkhälsan Research Center, Helsinki, Finland; 8 Terveystalo, Kuopio, Finland; 9 Varkaus Hospital, Varkaus, Finland; 10 Suonenjoki Health Centre, Suonenjoki, Finland; 11 Pohjola Hospital, Kuopio, Finland

**Keywords:** Seropositive rheumatoid arthritis, Epidemiology, Incidence, Public health

## Abstract

The objective is to evaluate the incidence of seropositive rheumatoid arthritis (RA) over 40-year period in Northern Savo, Finland. Data on new seropositive RA patients according to the American College of Rheumatology (ACR) 1987 classification criteria were collected in 2020–2021. In 2020 data on tobacco exposure, patient-reported dental health and living in residences with fluoridated tap water were gathered. Incidence rates were estimated and age- and gender-adjusted to Northern Savo population. The results were compared with data acquired in studies from 1980, 1990, 2000, and 2010. In 2020, 46 seropositive RA patients (21 females and 25 males) were recorded. The crude incidence of seropositive RA fulfilling the ACR 87 criteria in 2020 was 22.3 (95% CI 16.3 to 29.8)/100 000 and age and gender-adjusted 22.3 (95% CI 15.9 to 28.8)/100 000. Tobacco exposure > 5 pack years occurred 18% of females and 56% of males. Only 16% of males were full dentate. A total of 242 incident seropositive RA (age ≥ 16 years, 55% females) were identified in all study years. No differences in the gender-specific incidence rates in each cohort or in incidence between the studies every 10 years were recorded. The incidence of seropositive RA decreased in the age group < 55 years, *p* = 0.003. There was no change in the incidence of seropositive RA between genders or the study years. A declining trend for occurrence of seropositive RA in the young and early middle-aged population may reflect change in risk factors.

## Introduction

There is a growing body of epidemiological literature which recognises a temporal and geographic variability in the occurrence of rheumatoid arthritis (RA) in diverse populations.

The prevalence of RA has been 0.4–0.8% in Northern European countries and the annual incidence lies between 20 and 40/100 000 [1]. In Caucasian populations the incidence of seropositive RA has been declining [2, 3]. The incidence of rheumatoid factor (RF)-positive RA decreased in Finland between 1980 and 2000. The decrease was most substantial in the young and middle-aged, and it occurred in both genders [2]. A study from US showed that the total incidence of RA was stable during years 2005–2014 compared with the previous decade. However, the incidence of RF-positive RA decreased, whereas the incidence of RF-negative RA increased [3]. In the Pima Indian population, the prevalence of RF has decreased from 1966 to 1975 to more recent decades in both genders [4].

Smoking is known to predispose especially to RF-positive and anti-citrullinated protein antibody (ACPA)-positive RA [5, 6]. Yet, it was shown to increase the risk of developing both ACPA-positive and negative RA with a threshold of 2.5 and 5 pack years, respectively. In addition, duration of smoking seems to have a higher influence on evolution of RA than intensity of smoking. Despite the number of cigarettes per day, those who had been smokers for over 20 years had about a threefold likelihood of ACPA-positive RA and a 60% increased probability on developing ACPA-negative disease [6]. Even early life passive exposure to tobacco smoke might influence the risk of RA, at least in future smokers [7, 8].

In parallel, periodontitis has been linked to chronic diseases, such as RA [9]. Of periodontal bacteria, *Porphyromonas gingivalis* (*P. gingivalis*) and *Aggregatibacter actinomycetemcomitans* (*A. actinomycetemcomitans*) have been suggested to influence the pathogenesis of RA through protein modifications. *P. gingivalis* citrullinates proteins via the endogenous peptidylarginine deiminase enzyme, whereas *A. actinomycetemcomitans* induces hypercitrullination in neutrophils by activating human peptydylarginine deiminase enzymes through the actions of leukotoxin A [10, 11].

There is some evidence that fluoride in drinking water in a certain concentration prevents not only caries, but also periodontitis [12–15].

Aim of the study was to demonstrate trends in the incidence of seropositive RA in Northern Savo during 40-year period.

## Materials and methods

### Present study

The Northern Savo 2020–2021 study covered all rheumatological outpatient clinics, hospitals and rheumatological private practices (years 2020–2021), where patients with rheumatological diseases are treated in Northern Savo area. The population aged 16 years or over in the area is about 206 000 inhabitants, and a third comes from Kuopio City. Kuopio was the only Finnish community, where fluoridation of drinking water was carried out in 1959–1992 to reduce the incidence of dental caries. The average fluoride content of the Kuopio public water supply was 1.0 mg/l [15].

### Patients

All adult patients (age ≥ 16 years), who were registered in the Northern Savo area, and had an undiagnosed and untreated inflammatory joint disease from 1^st^ January 2020 to 31^st^ December 2020 were evaluated. Because of potential problems due to COVID-19 infection, data collection was continued to the end of 2021 to confirm the results of the 2020 study. Those patients who had a traumatic condition, purulent arthritis, connective tissue disease, vasculitis, viral arthritis, previously diagnosed osteoarthritis, crystal-induced arthritis, only tenosynovitis or bursitis were excluded.

### Clinical data

Data on age, gender, symptom duration, dental health, smoking, and years in residences with fluoridated tap water supply between years 1959–1992 (Kuopio City) were collected. Smoking was categorized into two classes by pack years and exposure time. Furthermore, dental status was classified as follows: full dentate, missing teeth, part denture or edentulous. On the first visit tender and swollen joint counts (out of 66/68 joints) were recorded. Data on erythrocyte sedimentation rate, C-reactive protein (CRP), RF and ACPA were recorded. Radiographs of hands and feet were taken. The diagnosis was established after the first visit according to clinical, laboratory, and imaging data available for or ordered on initial visit. The incidence date was the date of diagnosis. A patient was considered to have RA if he/she met at least 4 of the American College of Rheumatology (ACR) 1987 classification criteria [16]. Fulfilling the criteria of seropositive RA, the patient had to have either elevated level of RF or ACPA or both. Later case ascertainment data were reviewed by PE and OK-S. Radiographs were missing for two patients at diagnosis. As the study was cross-sectional, there was no follow-up for the patients not satisfying the inclusion criteria on the first visit.

### Other epidemiological studies

Epidemiological data have been collected earlier in several studies from years 1980, 1990, 2000, and 2010 [2, 17, 18]. Drug reimbursement certificates from new patients who were entitled to the special drug reimbursement to RA in the Finnish Sickness Insurance Scheme in 1980, 1990, and 2000 were collected through the registry of the Social Insurance Institution and evaluated for the diagnostic category as described earlier in more detail [2, 17]. Data concerning the fulfillment of the ACR 1987 classification criteria for RA and demographic data on the patients were obtained from the drug reimbursement certificates (1980, 1990, and 2000) and from the study files (2010). The incidence date was the date of entitlement for special reimbursement for RA medication for cases in 1980, 1990, and 2000 cohorts and the date of diagnosis in 2010. The delay from the diagnosis to grant of reimbursement decision is nowadays less than 1 month. Information on the age distribution of the population in the study area on those years was obtained from Statistics Finland [19].

### Epidemiological data analysis

Categorial data are given in numbers and percentages and continuous variables as means with standard deviations. Independent sample *t* test was used for comparison between two independent groups and analysis of variance (ANOVA) between five groups and the chi-squared test for comparison categorial variables. Crude and age-adjusted incidence rates were given for each cohort. Ninety-five per cent confidence intervals (95% CI) for the incidence rates were calculated using the Poisson distribution. The incidence rates were age- and gender-adjusted to the population in Northern Savo. The significance between the rate of incident cases and the time period was tested by the Mantel–Haenszel test for linear trend. A general linear model (Poisson link) was used to evaluate the relationship between the adjusted incidence rates by gender.

### Ethics

The studies were performed according to the principles of the Declaration of Helsinki and approved by the Ethics Committee of the Kuopio University Hospital (75/2001, 127/2009 and 1689/2019) and the Rheumatism Foundation Hospital (1991). All patients included in the studies 2010 and 2020–2021 gave a written consent. Two persons in both 2020 and 2021 denied participating in the study. A permission of the Ministry of Health to use the drug reimbursement certificates and the permission of the Social Insurance Institution to obtain the copies of the drug reimbursement certificates were required for data from years 1980, 1990, and 2000.

## Results

In 2020, 46 adult incident RA patients (21 females and 25 males, mean age (SD) 65.6 (14.3) years), were recorded. The result was confirmed with 46 (22 female and 24 male) registered patients in 2021. The crude incidence of seropositive RA according to the ACR 87 criteria in 2020 was 22.3 (95% CI 16.3 to 29.8)/100 000 and age- and gender-adjusted 22.3 (95% CI 15.9 to 28.8)/100 000.

**Fig. 1:**
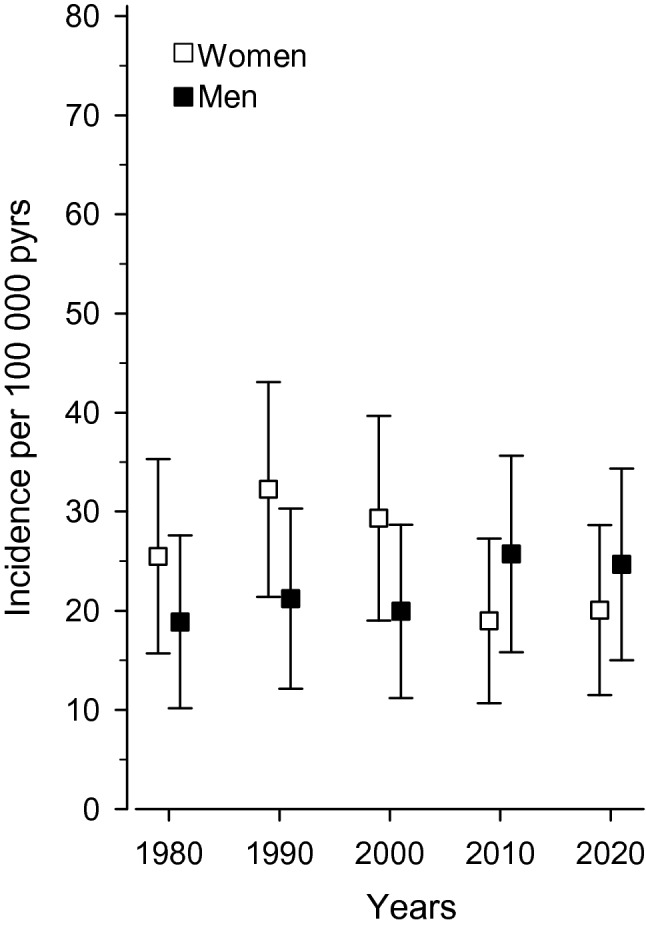
Age-adjusted incidence rates of seropositive rheumatoid arthritis according to the ACR 87 criteria among women and men in Northern Savo in 1980, 1990, 2000, 2010 and 2020

**Fig. 2:**
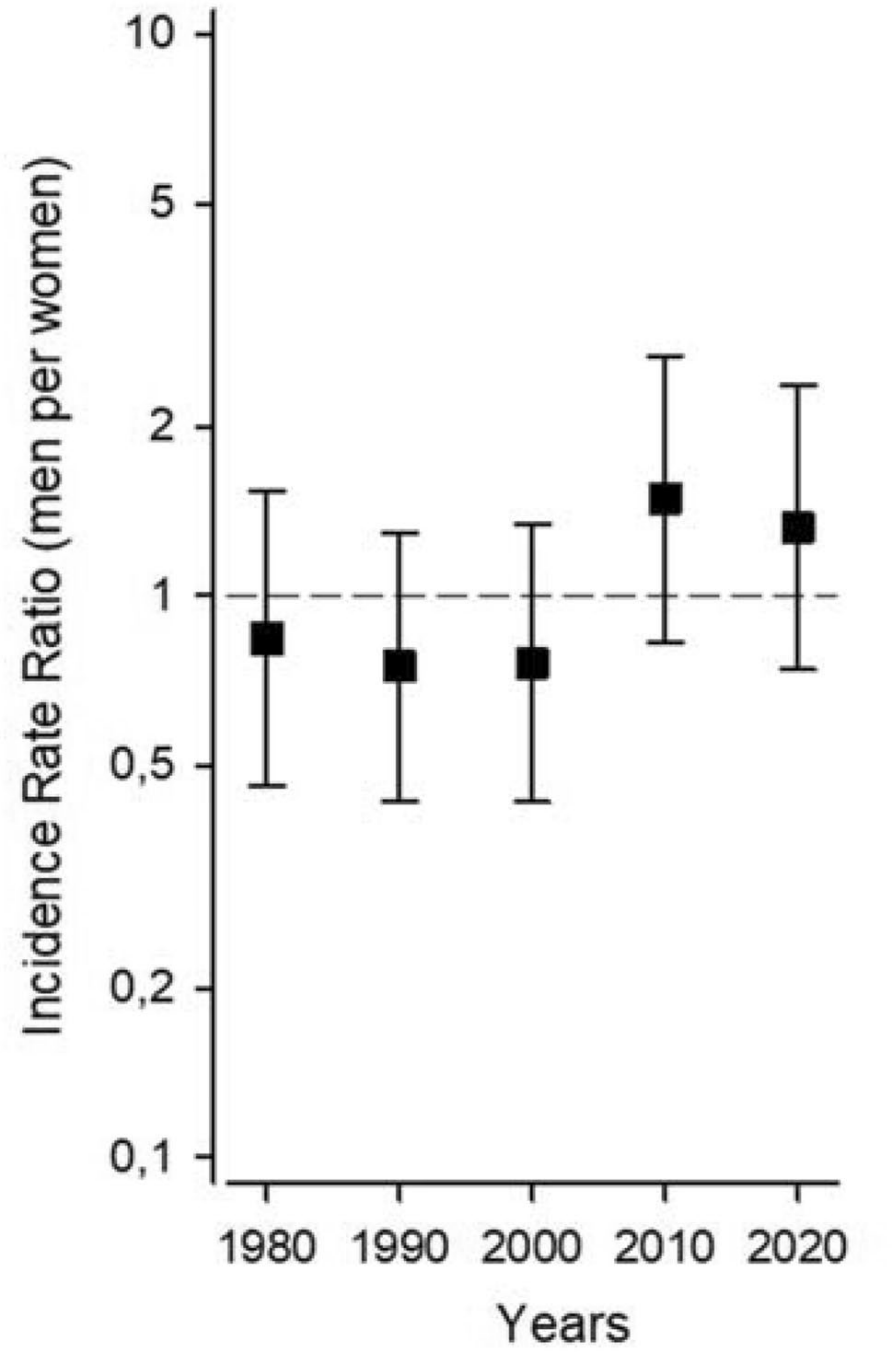
Age-adjusted incidence rate ratios (men per women) of seropositive rheumatoid arthritis according to the ACR 87 criteria in Northern Savo in 1980, 1990, 2000, 2010 and 2020

Seropositive RA was diagnosed in 242 adult subjects (132 females and 110 males) in 1980, 1990, 2000, 2010, or 2020. The clinical characteristics of the study population are displayed in Table 1. The mean age at diagnosis increased in both genders across the study years, but between genders no differences were recorded. The mean number of fulfilled ACR 1987 criteria fluctuated between the cohorts, but the proportion of patients with erosive changes at diagnosis declined about 50 percentages. The incidence rates among females and males are shown in Table 2 and Fig. 1 and age-adjusted incidence rate ratios males per females in Fig. 2. No differences in gender-specific incidence rates in each cohort or between the study years were recorded.

**Table 1:** Clinical characteristics of the patients with seropositive rheumatoid arthritis according to the ACR 87 criteria among females and males in Northern Savo in 1980, 1990, 2000, 2010 and 2020

Year	1980	1990	2000	2010	2020	*p*
Patients (*n*)	44	55	51	46	46	
Mean age at diagnosis (years, SD)	51.0 (12.4)	56.6 (15.4)	56.8 (14.9)	61.6 (12.3)	65.6 (14.3)	< 0.001
Females (*n*, %)	26 (59.1)	34 (61.8)	31 (60.8)	20 (43.5)	21 (45.7)	
Mean age at diagnosis (years, SD)	48.9 (14.5)	54.2 (17.4)	55.8 (17.9)	64.8 (13.8)	65.3 (14.5)	0.002
Males (*n*, %)	18 (40.9)	21 (38.2)	20 (39.2)	26 (56.5)	25 (54.3)	
Mean age at diagnosis (years, SD)	54.2 (7.7)	60.3 (11.0)	58.5 (8.8)	59.1 (10.5)	65.8 (14.4)	0.016
Mean number of fulfilled ACR1987 classification criteria (SD)	4.73 (0.73)	5.07 (0.86)	4.71 (0.67)	4.54 (0.72)	4.67 (0.67)	0.006
Erosive disease at diagnosis (%)	23 (71.9)	32 (71.1)	26 (52.0)	14 (35.9)	17 (38.6)	< 0.001
Data on radiographs available (*n*, %)	32/44 (72.7)	45/55 (81.8)	50/51 (98.0)	39/46 (84.8)	44/46 (95.7)	

*n* number, *SD* standard deviation, *ACR* American College of Rheumatology

*p* < 0.05 is regarded significant

**Table 2:** Annual crude and age-adjusted incidence rates per 100 000 person years of seropositive rheumatoid arthritis according to the ACR 87 criteria with 95% confidence intervals among females and males in Northern Savo in 1980, 1990, 2000, 2010 and 2020

Study year	1980	1990	2000	2010	2020	*p* for trend
Female patients (n)	26	34	31	20	21	0.12
Crude incidence 95% CI	25.5 (16.7 to 37.4)	32.2 (22.3 to 45.1)	29.3 (19.9 to 41.6)	19.0 (11.6 to 29.3)	20.1 (12.4 to 30.7)	
Age-adjusted incidence 95% CI	25.5 (15.7 to 35.3)	32.2 (21.4 to 43.1)	29.3 (19.0 to 39.7)	19.0 (10.7 to 27.3)	20.1 (11.5 to 28.6)	
Males patients (n)	18	21	20	26	25	0.28
Crude incidence 95% CI	18.9 (11.2 to 29.8)	21.2 (13.1 to 32.4)	19.9 (12.2 to 30.8)	25.7 (16.8 to 37.7)	24.7 (16.0 to 36.4)	
Age-adjusted incidence 95% CI	18.9 (10.2 to 27.6)	21.2 (12.1 to 30.3)	19.9 (11.2 to 28.7)	25.7 (15.8 to 35.6)	24.7 (15.0 to 34.3)	

*ACR 87 criteria* American College of Rheumatology 1987 classification criteria. *N* number, *CI* confidence interval. Age-adjusted to Northern Savo 2020 population. *p* < 0.05 is regarded significant

**Fig. 3:**
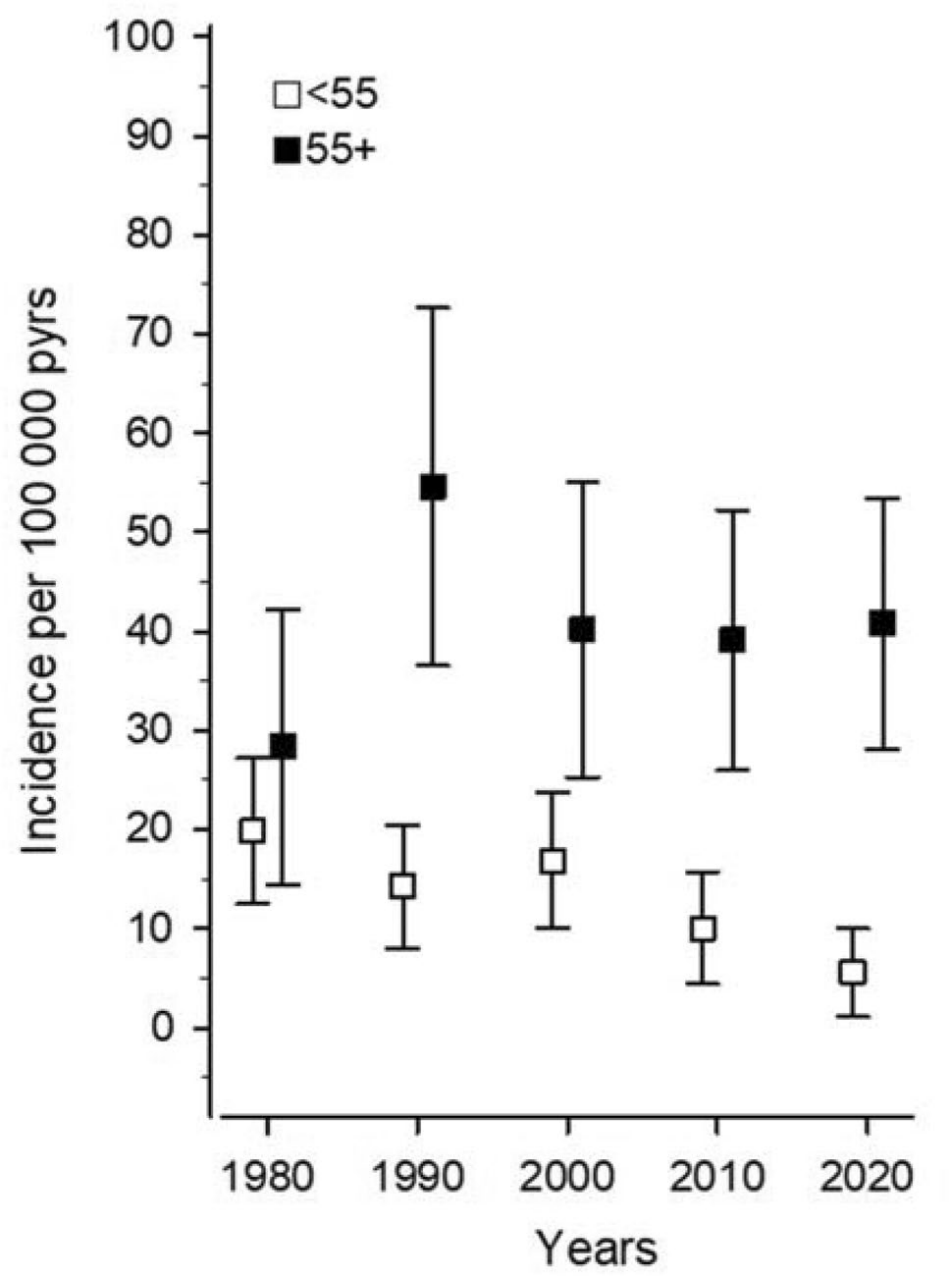
Age-specific incidence of seropositive rheumatoid arthritis according to the ACR 87 criteria in the age groups under and over 55 years in Northern Savo in 1980, 1990, 2000, 2010 and 2020. In the age group < 55 years *p* for linearity is 0.003

The incidence rates of seropositive RA fluctuated in the early cohorts between 28.4 (95% CI 14.5 to 42.3) and 54.6 (95% CI 36.5 to 72.6)/100 000 but stabilized to the level 40.2 (95% CI 25.3 to 55.1)/100 000 in the age group over 55 years. The incidence rate decreased from 19.9 (95% CI 12.5 to 27.2) to 5.6 (95% CI 1.1 to 10.0)/100 000 in the age group under 55 years during the study period (p for linearity 0.003) as shown in Fig. 3.

Tobacco exposure over five pack years occurred in 4/21 (18%) of females and 14/25 (56%) of males in the 2020 cohort. Two additional males (8%) were exposed over 10 years with smaller amount of pack years. Two females and four males were current smokers. All patients aged under 55 years had tobacco exposure. Among 46 patients only four male patients reported full dentate, whereas 7/21 (33%) of females and 4/25 (16%) of males were edentulous, and 1/21(5%) female and 10/25 (40%) male patients had part dentures. One female (5%) and eight male patients (32%) reported living in residences with fluoridated tap water for over 10 years. Of them, six (75%) males had significant tobacco exposure.

## Discussion

The present work demonstrates the changes in the occurrence of seropositive RA in a defined area using the same classification criteria over 40 years. In our series no trends in the gender-specific incidence rates of seropositive RA were recorded. However, the age at diagnosis increased in both genders about 15 years and the proportion of patients who had erosions at diagnosis decreased. The incidence declined in the age group under 55 years. Interestingly, there was no female predominance in incidence rates. Both genders reported marked teeth problems (over a third of the cases), but men had more significant tobacco exposure. Case ascertainment among women from the residents with earlier fluoridated tap water was lower than assumed to be based on their proportion in the study population.

The latest results are in accordance with the previous findings from the area with about one million adults including also Northern Savo population. In the aforementioned study we found a declining trend in the total and gender-specific incidences of seropositive RA.

The age-specific incidence rates decreased in the 35–54 year age groups in females and the 45–54 year age group in males. In the present study on a population of one fifth of that in the earlier study we could not show the declining trend in the gender-specific incidence rates, but a decrease in the incidence occurred in the same age groups as previously were reported [2].

The conventional ratio 2:1 between women and men in the occurrence of RA could not be verified, which might result from diverse risk or protective factors [2, 3, 6, 9, 20]. In the 2000–2014 Finnish nationwide series, in which the diseases were classified according to the ICD-10 codes used for granting drug reimbursements, the incidence in women was twice that in the present study but in men no differences were detected [20]. The traditional female predominance in RA was also seen in the Norfolk Arthritis register study in the UK in 1990 and in a Swedish prospective population-based study from years 1990–2000 [21, 22]. Moreover, there are number of European population-based register studies on incidences in Sweden, Norway, Denmark, and Italy, which all confirm traditional sex distribution in RA [23–26]. In a Danish study no changes were recoded between the earliest and latest study cohorts, as the proportion of women was 70% in 1998–2004 and 67% in 2012–2018 [25]. In Italy the female/male ratio was 3.5 before the fifth decade of life, but it decreased to 1.1 in the ninth decade of life [26]. In the above-mentioned Norfolk register incidence of RA was the highest in 45–74-year-old women, whereas in men it increased with age and the highest rates were seen in men over 65 years of age [21]. The incidence rate peaked at 70–79 years of age in both women and men in Sweden, Norway, Denmark, and Italy which could indicate that the highest incidence of RA has changed into the older age class [23–26].

Among Finnish men smoking has declined considerably during the last decades. In the 1970s half of the men were daily smokers, whereas in 2020, the smoking rate was 14%. In addition, women have cut back on smoking. During 1980–2010 about a fifth of women smoked regularly, while in 2020, only 11% of Finnish women were habitual smokers [27–29]. In the present study the proportion of current smokers was at the same level as in the general population. Yet, two-thirds of the male patients had a significant lifetime exposure to tobacco.

A meta-analysis of 14 studies showed that smoking raises the likelihood of periodontitis by 85% [30]. Dental health in Finland has improved between two studies 20 years apart from 1980 to 2000 [31, 32]. In the same time frame, the occurrence of untreated dental caries in the Finnish patients has diminished from 61% [31] to 31% [32]. In a later follow-up study severe periodontal pocketing was observed in 18% of women and 28% of men [33]. In 2011, a fifth of over 65-year-old persons were edentulous [33]. Moderate periodontitis was reported in 67% of the early RA patients and in 40% of the population controls in a Finnish prospective follow-up study from years 2005 to 2014. In addition, other periodontal findings were seen significantly more frequent in early RA patients than their counterparts [34]. In the present study the percentage of edentulous persons was higher in female patients with seropositive RA (29%) than in the population and about 40% of the patients in both genders reported significant loss of teeth.

Salivary carriage of periodontal pathogens seems to be high in the Finnish population. At least one pathogen was detected in 88% of the 1294 southern Finnish study participants (aged > 30 years) in years 2000–2001 [35]. In two Finnish studies the occurrence of *P. gingivalis* associated with age; The higher the age of patient, the higher the occurrence of that bacteria [18, 35]. In the present study the incidence of seropositive RA decreased in the age group under 55 years. Coronary heart disease (CHD) share risk factors with seropositive RA, and an approximately 80% decrease has been reported in the mortality of CHD in both genders in the middle-aged (35–64 years) population in 40 years [36]. Of the known risk factors, the prevalence of *P. gingivalis* and exposure to tobacco, are lower in these than in the older age groups [29, 35].

In an Indian study, the level of periodontitis was inversely related to the fluoride levels in drinking water [13]. Correspondingly, a Canadian study showed a positive correlation between fluoridation of drinking water and oral health status. During fluoridation of drinking water, one-third of the population of Northern Savo lived in Kuopio City [37]. In the present study fewer female than male RA patients reported living in residences, which were supplied with fluoridated tap water. Assuming that the incidence of RA was equal in the whole study area, the incidence among females who had been living in residences with fluoridated tap water should have been higher. The gender difference in seropositive RA has been conventional in the beginning of this millennium in a series on the whole Finnish population [20], and in the US, Minnesota [3]. It would be tempting to assume that women got extra protection against RA from fluoridation, whereas men lost their advantage owing to higher frequency of smoking. However, due to cross-sectional nature of the present study and low number of patients, conclusions of the causality are not allowed.

The disappearance of gender difference in RA patients suggests that there are possible protective factors in women`s life, which cannot totally be abandoned. Some evidence propose that the use of oral contraceptives may lower the likelihood of developing RA. The protective result was seen especially against RF-positive RA [38]. Similar results have been associated with long-lasting lactation [39].

The present study has the strengths of periodic collection of incidence data of seropositive RA using the same classification criteria in a defined area over 40 years. Quite stable numbers of incident cases in each study year support the assumption that no bias in catching patients occurred. Despite the start of COVID-19 epidemics in spring 2020, the number of seropositive RA cases did not significantly differ in years 2020 and 2021 from those in the earlier study years. The sensitivity of gaining entitlements for medication as a method for identifying patients needing long-term therapy has shown to be 95% in a prospective incidence study [40].

In these cross-sectional studies the limitation is the small number of annual cases. In addition, the proportion of population which was exposed to fluoridated tap water can only be estimated. By taking migration in the area into account, it can be estimated that about a third of the adult population aged 40 years or over in Northern Savo has been exposed to fluoridated drinking water at least for 10 years. The results of the present study are not generalizable as such to elsewhere due to different methods to improve public health in the studied area.

In the present study no difference between the genders in the incidence of seropositive RA was seen. There was a declining trend in the incidence of seropositive RA in the young and middle-aged, which may reflect changing of risk and protective factors. However, these observations need to be confirmed with a prolonged follow-up in a defined area and studied in a different research frame in future.

## Data Availability

The datasets generated during and/or analyzed during the current study are not publicly available due to confidentiality issues. Supplementary data are available from the corresponding author on reasonable request.
